# Psychological Screening for Exceptional Environments: Laboratory Circadian Rhythm and Sleep Research

**DOI:** 10.3390/clockssleep2020013

**Published:** 2020-04-15

**Authors:** Stephen A. Amira, Brenda L. Bressler, Jung Hie Lee, Charles A. Czeisler, Jeanne F. Duffy

**Affiliations:** 1 Division of Sleep and Circadian Disorders, Departments of Medicine and Neurology, Brigham and Women’s Hospital, Boston, MA 02115, USA; S.amira@comcast.net (S.A.A.); blb197@g.Harvard.edu (B.L.B.); jhielee@kangwon.ac.kr (J.H.L.); cacadmin@partners.org (C.A.C.); 2 Division of Sleep Medicine, Harvard Medical School, Boston, MA 02115, USA; 3 Graduate Program, Harvard Extension School, Harvard University, Cambridge, MA 02138, USA; 4 Department of Psychiatry, Kangwon National University School of Medicine and Center for Sleep and Chronobiology, Kangwon National University Hospital, Chunchon 200-947, Korea

**Keywords:** chronobiology, psychophysiological, selection criteria, mood

## Abstract

Selecting participants who constitute a representative sample while protecting them from potential adverse outcomes is a concern for clinical researchers. Our research group conducts deep phenotyping studies of the circadian timing system and sleep–wake regulation in long (up to 3 months) laboratory experiments, similar in many ways to “exceptional environment” conditions. Here, we describe the psychological screening process we have used for more than 30 years. We outline our “Select In” and “Select Out” measures within three major categories: psychological, psychophysiological, and psychosocial factors. We describe the screening process, inclusion–exclusion criteria on standard questionnaires, and clinical interview questions. We also describe how we manage the exclusion process during screening, ensure continued psychological health during the laboratory study, and manage study terminations. We present data from one recent study, outlining the number of individuals excluded at each stage of the process and present subjective mood data from the included individuals, showing the trajectory of mood across the five-week laboratory study and the end-of-study debriefing, during which the participants rated their comfort with various aspects of the study and their willingness to return for a future study. While designed for our inpatient research studies, elements of these procedures may also be useful for selecting individuals for other exceptional environments.

## 1. Introduction

The psychological response to the challenges of living and working in exceptional environments has been a subject of inquiry for decades. From space exploration to polar expeditions, screening and selection of individuals who will function well in confined spaces and relative separation from the outside world is critical for mission success. Our investigators in the Division of Sleep and Circadian Disorders have been studying individuals in long-term (up to 90 days) laboratory studies of circadian rhythmicity and sleep for more than 40 years. Some features of the study protocols include: living in a time-free environment (no access to information about time of day or day of week); restriction to a single hospital room and adjoining bath for the duration of the study (ranging from a few days to nearly three months); monitoring and collection of numerous physiological measures, including blood, saliva, urine, and stool samples; monitoring electroencephalography (EEG), electrooculogram (EOG), and electrocardiogram (EKG) overnight to determine sleep status, as well as during waking; collecting continuous recordings of skin and core body temperature (the latter via a rectal thermistor); special assessment procedures that can involve long periods of wakefulness while restricted to bed; exposure to varying lighting conditions, including very dim or very bright light; manipulation of sleep duration (both shorter and longer than typical) and day length (scheduling participants to live on non-24-h schedules); and other interventions and conditions; all in effect constituting what has been called generically, an “exceptional environment” [1].

### 1.1. Background

Much of what we know about human response to relatively separated and confined environments comes from studies done at the Antarctic expeditionary stations over the last century [2,3]. These studies have identified challenges and characteristics that apply not only to the Antarctic, but also have had applicability to space missions [4,5], the submarine service, and Sea Lab [6]. Even though volunteers in our experiments (unlike crew members in an Antarctic, space or submarine mission) are free to discontinue their participation in the experiment at any time and rejoin the outside world, we believe the challenges and characteristics they face may be relevant to those other exceptional environments. For the prior applications, psychological screening basically focused on two points as described by Palinkas [2]: (a) to “Select Out” or disqualify anyone with a psychological condition and/or psychiatric disorder, current psychological symptoms, or other risk factors (e.g., history of incarceration, former political prisoners, those suffering from PTSD); and (b) to “Select In” or find individuals with characteristics predictive of optimal performance and the ability to comply with the requirements of the mission [2].

A comprehensive summary of the challenges typically encountered in exceptional environments may be found in Leach [1] and includes three major categories: psychological, psychophysiological, and psychosocial factors. In our studies, the most relevant concerns include restrictions in activity level due to the laboratory environment, the absence of time cues, periods of acute and/or chronic sleep loss, variations in light intensity and exposure, extended episodes of restriction to bed rest, a loss of a sense of autonomy due to frequent monitoring and precise scheduling, and limited communication with family and friends during the study (see Table 1). 

In other exceptional environments, as noted in a NASA evidence report, the potential negative reactions to such demands have been cataloged and include cognitive decline (attention, concentration, reasoning ability), emotional upset, interpersonal conflict, and physical symptoms [7]. 

From the very beginning of our efforts to select participants for our research studies we have had to consider psychological factors, namely, how to select participants who are most representative and how best to protect participants from any adverse effects that might result from these exceptional study conditions. While we are well aware that our laboratory conditions may qualify as an “exceptional environment”, it should be emphasized that it differs significantly from the other exceptional environments mentioned previously, in that, unlike astronauts in space or scientists overwintering in Antarctica, our participants always have the option of withdrawing from the study at any moment. Moreover, our prior awareness of the psychological reactions to those other exceptional environments led us to build into our protocol as many protective elements as possible in order to shield against adverse reactions. Below, we describe what we have learned so that others may benefit from our experience as this research field continues to move forward.

With the aforementioned in mind, our challenge has been to obtain the most authentic representative sample of participants as possible for generalizable results while excluding those whose physical or psychological health would be negatively impacted by participation in a study (See Table 2). We and our institutional review board have been mindful of the level of compensation to avoid overly influencing a participant’s informed consent (i.e., economic coercion [8,9], as well as the consideration that potential participants may either consciously deny or simply not appreciate the nature of their own or their relative’s psychological conditions or diagnoses that might exclude them from participation.

While the detailed screening procedures described below were designed specifically for our inpatient sleep and circadian rhythm studies, they are based on the literature of selecting personnel for exceptional environments and therefore may be useful in selecting participants/operators/employees for other exceptional environments.

### 1.2. The Psychological Screening Process

During a National Institutes of Health (NIH) site visit of our program many years ago, the review committee expressed concerns as to what steps we would take to ensure the safety of the participants while achieving the stated goals of our studies given the evident demands incurred. They agreed with our proposal for the following: Participants with a history of significant psychiatric or psychological conditions should be selected out. Such conditions include major depressive disorder, bipolar disorder, schizophrenia or other psychoses, obsessive compulsive disorder, panic and other anxiety disorders, alcohol and drug abuse, developmental disorders, as well as attentional problems (e.g., attention deficit hyperactivity disorder). Furthermore, if any participant had been psychiatrically hospitalized and/or prescribed psychoactive drugs, neuroleptics, mood stabilizers, stimulants, or anxiolytics they should be excluded, even if the necessity of those were called into question by the participant. We recognized that limitations in time and access to past medical records would, in most cases, preclude a current in-depth investigation of the accuracy of such past circumstances. We, therefore, determined that we would yield to the clinical judgment of the past care provider in all cases.

We also made the decision that if any of the adult participants’ first-degree relatives had any of the aforementioned conditions (excepting addiction), the participant would also be excluded. This was based on the idea that many psychiatric disorders have a genetic component, and if that were the case the participant could be predisposed to the condition, therefore the potential stress of taking part in a study could precipitate the emergence of the condition. We based this decision on reports that when a first-degree relative has one of the major psychiatric disorders, the likelihood of the individual also having one of those disorders goes from 1–5% in the general population to 10–15%, and to approximately 50% if both parents have one of the aforementioned disorders [10]. A participant’s or relative’s psychotherapy or counseling in and of itself is not necessarily an automatic exclusion, so long as there was no concomitant drug regimen, hospitalization, or significant impairment.

In addition to psychological and family history, we also evaluate each potential participant with a psychological screening battery consisting of: the Minnesota Multiphasic Personality Inventory-2 (MMPI-2 [11]), administered by a research assistant to serve as a first-line screen prior to further psychological inquiry; Symptom Checklist 90-Revised (SCL-90-R [12,13]); Beck Depression Inventory-II (BDI-II [14]; or Geriatric Depression Scale, GDS [15] in individuals 65 or older); and the State–Trait Anxiety Inventory for Adults (STAI-AD [16]). For individuals over age 65, we also administer the Mattis Dementia Rating Scale (DRS [17]) and the Folstein test/Mini-Mental Exam (MMSE [18]) to rule out age-related cognitive impairments. Each is administered with specific cutoff scores to limit overreliance on subjective clinical judgment (see Table 3). 

Our “Select In” criteria include characteristics such as: resilience and flexibility (in school, work, and life situations the participant has been able to subordinate individual wants to fit the demands of the situation); adaptability and coping skills (the participant finds ways to occupy/entertain themselves through interests and hobbies); positive interpersonal relationships (evident in the session with the psychologist and across the lifespan, the participant has reciprocal relationships without constant demands for personal gratification or difficulty with authority figures); commitment (the participant has been able to successfully complete school and work efforts without frequent changes or interruptions).

Finally, we carry out a clinical interview with a licensed psychologist (see Table 5 for an outline of the key points addressed during the clinical interview) with the primary focus to confirm that the participant knows what he or she is volunteering for and to determine the appropriateness of any individual for the particular study for which they are being screened.

### 1.3. The Clinical Interview: Details and Specific Levels of Inquiry

A twelve-point approach has been used to screen several thousand prospective participants over the past 35 years. These twelve-points are embedded within the following four categories: (A) study overview, (1) introduction; (2) “How did you hear of our study?”; (3) “Why volunteer for something like this?”; (4) “What is this study all about? Can you describe briefly what we are researching?”; (5) describe the study in detail; (B) medical history, (6) review of their living situation and their past and current health status; (C) personal history, (7) “Where were you born and where were you raised?”; (8) “Thinking about the first 12 years of your life, in a sentence or two, how was that time for you?” “What was it like?”; (D) mental status, (9) behavior and personal appearance; (10) vegetative functions assessment; (11) mental health history; (12) outside interests (see Table 4 for details). 

While there is some redundancy between the clinical interview and the medical exam that is also part of the screening process, we have found this to be useful, as we have sometimes encountered inconsistencies in the participant’s report, whereupon further exploration of the inconsistency led to reconsideration or disqualification of the individual. The medical and psychological history of family members is also important because participants often gloss over or “normalize” behaviors or proclivities of their family members, either out of ignorance or in a conscious effort to be accepted into the study. The personal history inquiry helps us discern some aspects of an individual’s personality, the participant’s ability to relate in a give-and-take manner, and their capacity for empathy and caring.

### 1.4. Disqualification Procedures

Some potential participants are disqualified even before the clinical psychological interview (see Table 4), based on high scores on one or more of the MMPI-2 [11] scales. Even though they are forewarned that they may be eliminated in this manner, some participants express concerns when this occurs. We, therefore, permit a telephone or in-person consultation with the clinician to discuss the results and implications at no charge to the participant. In most instances, the disqualifying score does not indicate anything serious or life-threatening; nonetheless, we take seriously what might be a forewarning of some emerging major issue (see Table 3). 

If a participant is disqualified during the clinical interview, or because of high scores on the three screening questionnaires taken at the time of the interview, they are told immediately, with the reason explained in detail. Regardless of when in the process the exclusion occurs (see Table 5), the psychologist will advise them about follow-up treatment, if warranted, and provide information about where they might get assistance. 

Special procedures are followed if a potential participant endorses items reflecting suicidal ideation or intent on the MMPI-2 [11] or the BDI-II [14]. Research assistants administering those tests are trained to notify the psychologist immediately so that further assessment by the psychologist can be carried out.

### 1.5. Monitoring the Psychological Wellbeing of Participants Empaneled in a Study

The staff of our research teams and of the Center for Clinical Investigation are well acquainted with the potential psychological challenges confronting participants and are mindful of the need to provide a supportive, friendly, and professional environment for the participants’ well-being. In addition to the general ethos just described, we are aware that we have technicians, support staff, and trainees who cycle through on internships and work–study programs. We, therefore, have training modules whereby a senior staff member and/or a former study participant discusses the participant experience with new staff members, and our senior psychologist presents an in-service seminar to all staff members periodically to underscore the need to be mindful of the potential psychological stresses of being in a study, how to best deal with participants, and how to be aware of signs of distress. 

While they are empaneled in the study, a regular part of most protocols involves cognitive tests presented on a computer in their study room—tests of attention, throughput, short-term memory, reaction time, visual selection, and subjective rating scales (including a mood survey), are done typically several times each day throughout the study. Ongoing review of the mood survey ratings provides an indication if there is a change of consequence in the participant’s emotional states that might require attention. In addition, the research nurses working in the Center for Clinical Investigation (CCI) evaluate the participant’s status on a daily basis.

It is also a routine practice that for studies lasting longer than three weeks, there is an in-study visit from the psychologist to assess how the participant is faring under the study conditions (see Table 6 for in-study visit process). In some cases, the institutional review board (IRB) may require a visit for a shorter study or may require additional visits. In addition to the routine visit, the psychologist or a backup psychiatrist is always available for consultation with staff members or to make an in-person visit if the situation requires it.

### 1.6. Psychological Care of Dis-empaneled Participants

We make it clear in both our interpersonal interactions (specifically at the initial screening visit, at time of physical examination, at time of the psychological exam, during consent visit, upon admission to the laboratory) and in the written study documentation that the participant may elect to withdraw from the study at any time. There are also situations, including medical or psychological, when the investigators may choose to dis-empanel a participant from a study that is underway. Over the years we have had to dis-empanel a limited number of participants for psychological or behavioral reasons (see Table 5). In all such cases, we have offered follow-up psychological care. 

### 1.7. Emergency Protocol

If a participant becomes depressed, anxious, or even suicidal, the psychologist or attending psychiatrist is called in for a timely, in-study visit with the participant. At this point, the participant is typically dis-empaneled from the study. A further assessment is made to determine their suitability for discharge and to provide immediate support. Depending on the circumstances, a dis-empaneled participant may stay at the hospital until a safe release is feasible (for example, ascertaining the availability of a friend or family member to accompany the participant home). The dis-empanelment process also includes efforts to help the participant make a transition back to their pre-study schedule to better facilitate a comfortable discharge. If necessary, we also provide a form of after-care including a follow-up session with the psychologist and professional referral for any subsequent treatment that is necessary. We cannot, however, force a participant to have a follow-up visit if they refuse, unless we believe there is an imminent risk to their safety requiring further intervention. To our knowledge, no participant has suffered any adverse effects requiring more than a single follow-up session. It should be noted that in the few situations in which a participant has been dis-empaneled for psychological reasons, it was almost always because the participant consciously misled us or denied pertinent history that would have otherwise excluded them or raised concerns (See Table 5).

### 1.8. Repeat Participants

Over time we have had the opportunity to bring back participants who are willing to participate in a study again as their own controls or to participate in different studies. The advantage to us is knowing from their prior experience that they can tolerate the conditions; moreover, their willingness to return is also a testament to their relative satisfaction with how they were cared for during their initial study. Nonetheless, before starting a new study, the former participant must be formally evaluated to update their current situation and meet with the psychologist to have the psychological screening measures re-administered. An individual’s status as a repeat volunteer does not guarantee their inclusion in subsequent studies, as they may still be excluded based on the updated findings. 

### 1.9. Screening and In-Study Data from One Example Study

While we have not maintained a comprehensive cross-study tally over the past 35 years, we detail below our experience in a recent study of the number of individuals screened out, those who were dis-empaneled during the study for psychological reasons, and those who successfully completed. The experiment in question was a series of 39-day laboratory studies carried out between 2007 and 2012 [19,20]. See Methods below for details of the study. 

We recruited healthy participants between 18 and 30 years old as well as those between 55 and 70 years old. Our screening process was similar to that described above, beginning with a telephone or email description of the study and a series of questions about overall health status and sleep habits. Those who passed this initial phone screen were invited to begin the formal screening process. This began with a visit during which they completed the MMPI-2 [11] and the Beck Depression Inventory-II [14] or Geriatric Depression Scale [15], along with several other questionnaires focused on their medical history and sleep–wake habits. The clinical psychological interview was typically scheduled as the third screening visit, after a medical screening visit that included a physical examination, electrocardiogram, blood tests (comprehensive metabolic panel, complete blood count), and urinalysis. If the participant passed the clinical interview, the final screening visit was an overnight clinical polysomnogram to rule out undiagnosed sleep disorders. 

## 2. Results

**Figure 1:**
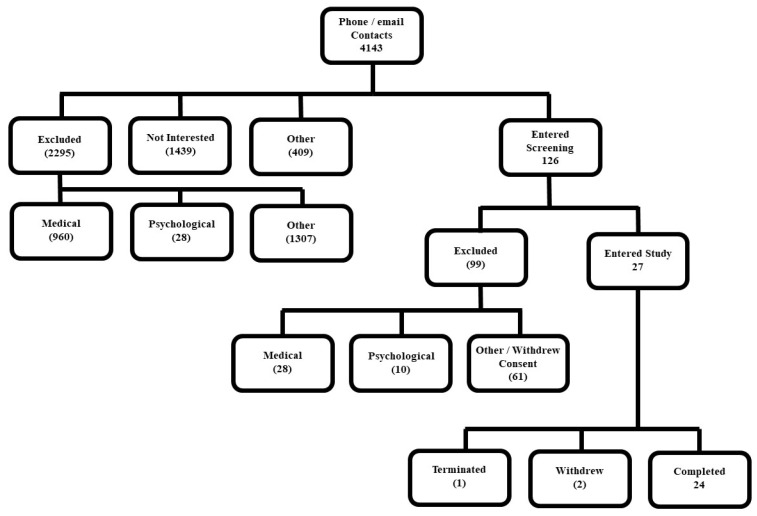
CONSORT flow diagram. The diagram shows participant exclusion and inclusion at different stages of the screening process for the representative study. The parenthetical values are the approximate number of participants excluded at various stages, whereas positive values (no parenthesis) represent inclusion at reflected stages.

As indicated in Figure 1, 27 individuals began the study—14 were in the older age group and 13 were in the young age group. Three participants did not complete the study. One young woman (age 26) dropped out of the study on day 12 after several days on the chronic sleep restriction-forced desynchrony (CSR-FD) schedule. She complained of nausea, diarrhea, and feeling cold and tired in the 1–2 days before ending the study. One older woman (age 58) dropped out of the study on day 7 while still in the baseline segment of the study. She had complained of skin irritation from the EEG electrodes, stomach discomfort, and pain at the site of her IV catheter, and expressed concerns that she would be unable to deal with the physical challenges of the study. 

The study of another older woman (age 55) was ended by the investigators on day 13. She had been having a difficult time adhering to the time constraints of the study schedule and learning to carry out the computer-based performance tests. Over several days, she became increasingly irritable and visibly frustrated with the pace of the study, and her inability to adapt to the study resulted in missed data. The psychologist was called in to assess the situation and meet with the participant. The participant noted that after two wake episodes she realized she was there seeking “self-mastery”. She expressed a litany of complaints and concerns: not enough time to eat at her pace, the air was too dry, she had problems with the temperature sensor, she was irritated when asked questions or having to adhere to the protocol schedule. She denied being upset but felt lectured to and scapegoated by the study staff. The psychologist explained that such studies are not right for everyone and thanked her for her participation, honesty, and feedback; however, he informed her that the study team needed to end her stay due to concerns for her comfort and well-being. 

A total of 24 participants completed the study (six young men, six young women, six older men, six older women). Our results from this study and others of similar duration are consistent with findings from other exceptional environments; despite screening, there are a small number of selected participants who will not adapt well. For example, despite stringent “Select Out” screening procedures utilized in the past in Antarctica, five percent of personnel still experienced significant symptoms of psychological distress [2].

### 2.1. Subjective Mood

**Figure 2:**
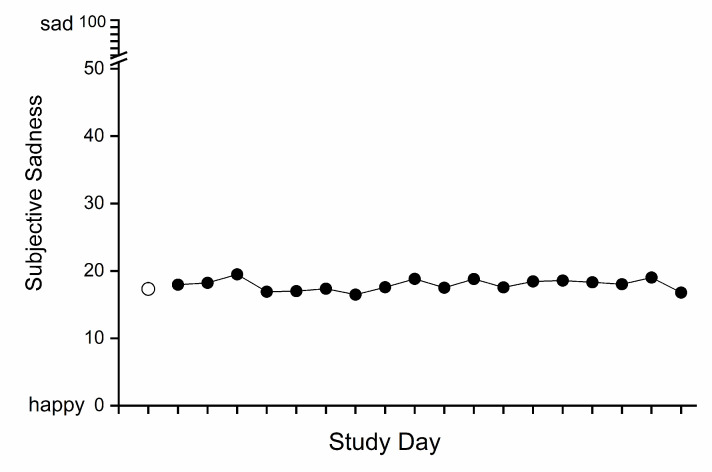
Effect of study segment (baseline vs. chronic sleep restriction-forced desynchrony (CSR-FD)) on happy–sad visual analog scale scores. The combined data from young and older participants are plotted for the baseline (○) and for each chronic sleep restriction-forced desynchrony (CSR-FD) day (●). The baseline mean (single point) is the average from the three baseline days. For each CSR-FD day, a daily average for each participant was first calculated and then those were averaged across participants.

Results from those participants who completed the study were included in the analysis of subjective mood. The mean scores (± SD) on the happy–sad scale (range 0–100) were 17.32 ± 13.07 for baseline days and 17.93 ± 15.57 for CSR-FD days. There was no significant effect of study segment (baseline vs. CSR-FD) on subjective sadness (F = 0.561, df = 3.76/86.52, *p* = 0.68, Greenhouse–Geisser corrected) in the 24 participants who completed the study, indicating that mood did not deteriorate over the three weeks of intervention when the participants were subjected to both chronic sleep restriction (equivalent to a 5.6 h sleep opportunity per 24 h) and chronic circadian disruption (see Figure 2). 

### 2.2. Overall Study Evaluation

**Figure 3:**
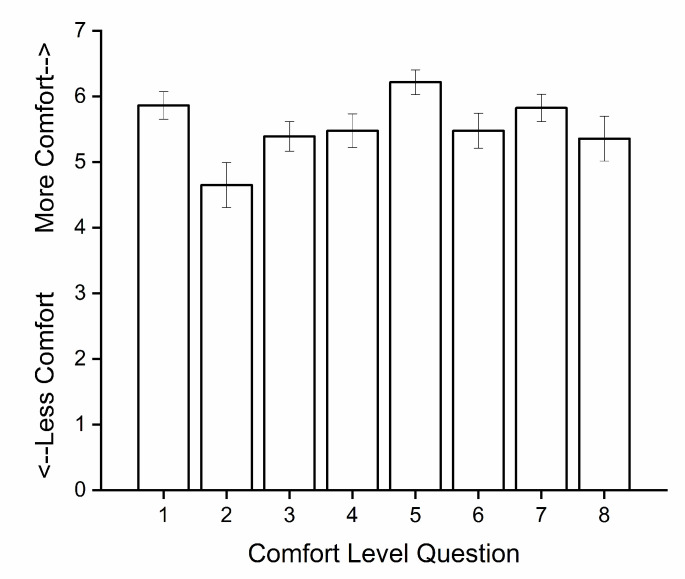
Subjective comfort level of study conditions evaluated at end of study. Each bar represents the mean and standard error of evaluations on one comfort level question from 23 of the 24 participants. Responses were rated on a 1 to 7 scale where 1 was “extremely bad” comfort level and 7 was “extremely good” comfort level. The comfort level categories shown on the x-axis are: 1: IV catheter, 2: core temperature sensor, 3: scalp electrodes, 4: face electrodes, 5: wrist activity monitor, 6: test frequency, 7: test duration, and 8: constant Posture condition.

As part of our discharge process, participants are asked to complete an exit questionnaire in which they give feedback on a variety of topics (see Methods below). Exit questionnaires were not collected from participants who did not complete the study. The exit questionnaire from one older (age 65) man who completed the study was missing, so data from the remaining 23 participants were included in the analysis. The mean comfort level (range 1–7) was 5.53 across all eight comfort categories surveyed and ranged from 4.65 (comfort of temperature sensor) to 6.22 (comfort of wrist activity monitor; see Figure 3). Twenty-one of the 23 participants (91%) responded positively to the question about whether they would be willing to come back for another study.

## 3. Discussion

In this paper, we have described the approach we use to screen participants for long laboratory phenotyping studies of circadian rhythms and sleep–wake regulation. Our successful screening approach is based on principles from “exceptional environment” [1] selection procedures and is focused on selecting in individuals who are representative and who will be successful within the constraints of the study, and selecting out individuals who would be at greater than normal risk (due to family history, personal history, personality type) for an adverse psychological reaction to the study conditions [2]. Those conditions are similar to many extreme or exceptional environments [1,2,3,6] in that the participant spends the study in a limited space (their study room); they are limited to mail contact with family and friends; they do not have control over many aspects of their daily life while in the study (sleep times, meal timing and content, free time); and they may experience physical discomfort due to the sensors used to monitor various aspects of their physiology. While the procedures were designed to select participants for our laboratory studies, they may have relevance for other situations in which individuals must be selected to operate in exceptional environments. 

## 4. Materials and Methods

The study was conducted in accordance with the Declaration of Helsinki, and the protocol approved in advance by the Partners Health Care Human Research Committee (2005-P-002292). Each participant provided written informed consent before participating. Investigators interested in data sharing should refer to Appendix A below.

### 4.1. Participants and Protocol

Healthy young (18–30 years of age) and older (55–70 years of age) participants were recruited for a 39-day laboratory study to examine age differences in the sleep, waking alertness/performance, and metabolic response to three weeks of chronic sleep restriction and circadian disruption [19,20]. Flyers posted on community bulletin boards, local newspaper advertisements, and online advertisements were used for recruitment. Participants had to be healthy, have no acute or chronic medical conditions, and not be regularly taking prescription or over-the-counter medications. Other requirements included a habitual sleep duration of 6–9 h each night, no major sleep complaints, no night shift work within the last year, and no travel across two or more time zones within the last three months. In addition to the psychological screening described above, each participant underwent a pre-study medical and sleep screening evaluation (see Figure 1). 

Prior to starting the laboratory segment of the study, participants were instructed to maintain a consistent sleep–wake schedule for at least 3 weeks, with 10 h per night scheduled time spent in bed at a self-selected time. Participants were then admitted to the Intensive Physiological Monitoring Unit of the Center for Clinical Investigation at Brigham and Women’s Hospital for the 39-day study. The first three days each included a 12-h nighttime sleep opportunity and a 4-h daytime nap opportunity. Days 4–6 included a 10-h nighttime sleep opportunity (referred to as ‘baseline’). This was followed by the chronic sleep restriction-forced desynchrony (CSR-FD) portion of the protocol, consisting of eighteen, 28-h “days” (equivalent to 21 calendar days), each consisting of a 21.47-h wake episode and a 6.53-h sleep opportunity (equivalent to 5.6 h of sleep opportunity per 24 h). Following this, a 10-day recovery segment was scheduled, during which participants had their sleep timing realigned to their baseline circadian phase and were given a 10-h sleep opportunity each night.

Throughout the study, research technicians observed participants during all waking episodes, either by closed-circuit television or direct observation within the study room. Waking EEG recordings were carried out to document vigilance state and polysomnography (PSG) recordings were carried out each night to record sleep. Light levels were maintained at <15 lux (<0.048 W/m^2^, dim indoor light) during wake episodes to avoid circadian phase-resetting effects of light, and all lights were turned off throughout the scheduled sleep opportunities. Major daily events including waketime, bedtime, mealtimes, and shower time were determined by the investigators. Participants were not allowed to get out of bed or turn on lights if they awakened before the scheduled sleep opportunity was over, and similarly were not allowed to lie down or nap during scheduled wake opportunities. 

### 4.2. Subjective Mood Data

During wake episodes, participants were given batteries of cognitive tests (to assess vigilance, throughput, visual selection, interference, short-term memory) every 2 h; those data will be reported elsewhere [20,21,22,23]. Subjective mood was assessed approximately once per waking hour using a linear, non-numeric, bipolar visual analog scale (VAS [24,25]) presented on a computer screen as part of a larger test battery. The scale was labeled “happy” at one end and “sad” at the other end, and the participant was instructed to indicate with a mouse click the point on the scale they were feeling over the past five minutes. Subjective mood data from the three baseline days and the 21 chronic sleep restriction-forced desynchrony (CSR-FD) days were analyzed using one-way repeated measures ANOVA to study the effect of study segment (baseline vs. CSR-FD) on the happy–sad scale scores. Significant effects were defined as *p*-values < 0.05. The data were analyzed using Package for the Social Sciences (SPSS) version 18.0 for Windows. 

In between major daily events and test batteries, the participants were allowed sedentary indoor activities such as writing, reading, board or card games, watching movies, arts and crafts, listening to or playing music, conversing with the study staff, and mild stretching (exercise was prohibited).

### 4.3. Overall Study Evaluation Data

On the final day of the study, after being informed that their study was complete and going through a discharge procedure, each participant was asked to complete an exit questionnaire. This questionnaire seeks feedback from the participant for the study staff (recruiter, project leader, research assistants) and CCI staff (technicians, nurses). It also seeks feedback about the study room, meals, and specific aspects of the study (equipment, procedures), using a 7-point Likert scale (from 1–extremely bad to 7–extremely good), as well as free text space to add comments. A final question about whether the participant would consider doing another study in the future is also included. Investigators and their study staff use this feedback to ensure the participant felt well-prepared for the study’s demands, and wherever possible, to make adjustments for future study procedures and briefings of prospective participants.

Our analysis focused on two aspects of the exit questionnaire data. First, for the quantitative ratings provided by all the participants, responses to each of the eight questions related to the study conditions were averaged across participants to calculate a mean and standard deviation for each question. Next, the percentage of participants who responded positively to the question about returning for another study was compiled.

## 5. Conclusions

We have described the psychological screening process we have used in our sleep and circadian rhythm research group over the past 30 years to select representative individuals for intensive inpatient studies. Our screening approach to selecting individuals is based on principles from “exceptional environment” [1] selection procedures. Our inclusion and exclusion criteria fall into three major categories: psychological, psychophysiological, and psychosocial factors. To most efficiently screen large numbers of participants, we use a mixture of standard questionnaires followed by a clinical interview. Once selected in, we have staff training processes and standard procedures to ensure the continued psychological health of each participant during the laboratory study. We also have standard procedures for excluding participants during screening, and for managing study terminations. 

As the study data we present for a very challenging 39-day study show, our procedures have overall been successful, with a very high percentage of participants completing the studies, saying they were satisfied with their study experience, and being willing to return to take part in a future study. While our procedures were designed to select participants for our laboratory studies, they may have relevance for other situations in which individuals must be selected to operate in exceptional environments. 

## Figures and Tables

**Table 1:** Brigham and Women’s Hospital Division of Sleep and Circadian Disorders Intensive Physiological Monitoring unit studies: psychologically relevant concerns.

**1. Restrictions in activity level** (e.g., light stretching only, no strenuous exercise, no formal meditation as this can drive metabolic output in two directions, therefore, people must be prepared to limit these activities as these are not variables we are testing) (e.g., no exercise or activities that change heart rate, no meditation, no lying down during the daytime)
**2. Absence of time cues** (e.g., no windows in study rooms; no clocks, watches, timers; no live television, radio, internet)
**3. Acute and chronic sleep loss and sleep distribution** **A.** Acute sleep deprivation ● e.g., >18 h continuous wake periods **B.** Chronic sleep deprivation: ● e.g., <7 h in a 24-h period over many days **C.** Sleep disruption: ● e.g., sleep scheduled at times when it is difficult to fall asleep or remain asleep
**4. Lighting intensity and exposure variations during sleep** **A.** Dim light (e.g., <4 lux) **B.** Bright light (e.g., >500–10,000 lux) **C.** Complete darkness (scheduled sleep episodes throughout)
**5. Prolonged sedentary behavior** **A.** Specialized circadian assessment—sitting in bed for extended time periods (e.g., procedures that last 16–50 h, no vertical position as it can impact blood pressure which is one of the time cues)
**6. Limited autonomy** **A.** Investigators monitor and control physiological functions according to precise schedules **B.** Timing and duration of meals and showers is limited by protocol demands **C.** Inflexibility of meal content once dietitians have prepared menu **D.** Frequent interruptions of free-time for study-related events
**7. Limited outside communication** **A.** No direct contact with friends, family, and significant others for the extent of their study **B.** Limited communication in the form of letters (no email, calls, etc.) **C.** Regular engagement with supportive trained staff only

**Table 2:** Five Select-Out/Select-In categorical criteria.

Five Select-Out/Select-In Categorical Criteria	
**1. Participant Biology**	To not skew or affect results such as neuroendocrine changes
**2. Participant Health Risks**	Where conditions may put them at risk to their physical health
**3. Participant Lab Risks**	Character types who are difficult to manage or who may be hostile and litigious.
**4. Participant Completion**	Those who are not likely to complete the study for physical or psychological reasons.
**5. Participant Incentives**	Due to cachet involved (for example, the desire to participate in a NASA-funded study) or level of remuneration offered

**Table 3:** Select-Out/Select-In testing measures.

Select-Out/Select-In Testing Measures	
**Minnesota Multiphasic Personality Inventory-2 (MMPI-2** [11]**)** (Administered by a research assistant to serve as a first-line screen prior to further psychological inquiry)	Depression scale, T > 70: excluded automatically
	Psychopathic deviance, T > 75: excluded automatically * schizophrenia, hypomania scales
	L, F, and K validity scales, T > 80: excluded automatically other clinical scales
	L, F, and K validity scales, T > 70: held pending interview ** and other clinical scales
**Symptom Checklist** **90-R** **(SCL-90-R** [12,13]**)**	Depression scale, Distress level >1.25: excluded automatically
	Hostility scale, >1: excluded automatically
	Phobic anxiety, >0.75: excluded automatically
	Paranoid ideation, >1.25: excluded automatically
	Psychoticism, >1: excluded automatically
	Anxiety, >1.25: excluded automatically
	Somatization, >1.00: excluded automatically
	Obsessive compulsive, >1.20: excluded automatically
	Interpersonal sensitivity, >1.25: excluded automatically
**Beck Depression Inventory-II** **(BDI-II** [14]**)**	Score of 10 or above excluded automatically
**State–Trait Anxiety Inventory for Adults (STAI-AD** [16]**)**	>40: excluded automatically
**Clinical Interview** **by Licensed Psychologist** **(see Table 4 for details)**	To confirm volunteer participant awareness and to determine individual study appropriateness
**Mattis Dementia** **Rating Scale** **(DRS** [17]**)**	<123: excluded automatically
**Folstein test/** **Mini-Mental Exam (MMSE** [18]**)**	<27: excluded automatically
**Geriatric Depression Scale** **(GDS** [15]**)**	10: excluded automatically

* Scores on all 3 of these scales are correlated with age; those below 26 years of age generally score higher on these scales, so we have adjusted our cutoff values accordingly. ** Certain subgroups (e.g., exchange students, emigres, etc.) with these scores naturally tend to score higher on these measures; they will be referred to Dr. Amira for clinical interview, and inclusion/exclusion will depend on the results of that clinical interview.

**Table 4:** The clinical interview: 12-point approach and levels of inquiry.

What Follows Here Is the Approach We Have Used for over 30 Years to Screen over Two Thousand Participants.
Interview Question(s)/Procedure(s)
● Outlines, Objectives, Rules, and Goals ✓ Points of Interest ○ Details and Side-notes
**Section A. Study Overview**
**1. An introduction:** **Helps put the participant at ease** **Takes some of the tension and mystery out of the process** ○ At-ease participant is far more likely to be forthcoming about critical information than a defensive one ✓ **Emphasize and inform participant of demanding rigor of lab screening** ✓ **Discuss study in detail ensuring full comprehension, and appropriateness of volunteer participation and requirements of all individuals**
**2.** ***“How did you hear of our study?”***
**3.** ***“Why volunteer for something like this?”*** ✓ **Remuneration needs to be acknowledged as a realistic primary motivation for taking on such a major commitment** ○ Typical responses that call for exclusion include: *“I am volunteering for self-mastery”, “I need the structure”,* and *“I am bored with my life,”*
**4.** ***“What is this study all about? Can you describe briefly what we are researching?”*** **Our research assistants inform volunteers what the purpose and demands of the study are before they get to the clinical interview** ✓ **Evaluate how well the volunteer assesses or acknowledges the possible challenges ahead** ○ Lack of understanding should be quickly rectified ○ Clinician should note participant’s reaction to the clarification
**5. We next describe the study in detail** **Participants must know the details and expectations:** ○ To satisfy the need for informed consent ○ For their own comfort and our benefit: discomfort, requirement uncertainty, and fears can be explored, allayed, or, alternatively, to allow them to withdraw from participation ✓ **Reviewed with them by the research assistant** ✓ **Outlined in the informed consent documents** ✓ **Reviewed again during the clinical interview:** ○ Physical lab conditions ○ Physiological functions to be measured ○ Limitations on some personal habits and activities (e.g., no strenuous exercise and no formal meditation) ○ Real limits to personal privacy (e.g., staff member access, cameras, etc.): ○ Ascertain compliance with the study conditions ○ Ask if there are any questions, worries, or concerns ○ Emphasize that there is no hidden agenda ○ Questions and appropriate information should be given with the exception of the correct time or date until the end of their study participation
**Section B. Medical History**
**6. Review of their living situation and their past and current health status** **6.A. Determine the circumstances of the participant’s current living situation, including co-habitants** ✓ **Have they any dependents who would suffer a hardship or about whom they would worry while in the study?** ○ The clinical psychologist may want to rule out those with significant others undergoing cancer treatments or coping with other physical challenges lest the participant worry while they are in the study **6.B. Have them designate one or two individuals who will be given 24-h emergency telephone numbers:** ✓ **Discuss the protocol we follow should there be an emergency in their outside life while they are in the study** ○ This has happened on several occasions (for example, on 9/11, when a family member of a participant died, or if someone has suffered a life-threatening injury, or there was a natural disaster such as a hurricane). See separate section here entitled Emergency Protocol **6.C. Explore the participant’s current aches or pains, any chronic medical conditions, any hospital admissions at any time in their lives:** ○ Confirmation step: as it is first addressed at the initial screening visit and again during the physical examination ✓ **They must list all medications, drugs, vitamins, nutrients, or supplements and must be willing and able (with their primary care physician’s agreement) to discontinue them (with some exceptions)** ✓ **Questions regarding breathing difficulties, palpitations, loss of consciousness, feelings of restlessness, agitation or panic, intolerance to particular foods, and chronic or recurrent gastrointestinal distress are all focused on discerning possible manifestations of anxiety disorders, phobias, and somatic forms of pathology** **6.D. Inquire about exercise—participants need to be able to tolerate the absence of any exercise regimen (especially if used for stress reduction) and must consider the likely loss of conditioning resulting from taking part in a long study:** ○ Serious athletes (e.g., marathoners, competitive, and semi-pro athletes) have either been disqualified or voluntarily withdrawn upon consideration of these restrictions. Regular gym goers are asked about steroid use and the use of other training supplements like creatine and whey protein **6.E. Participants are asked about their use of caffeine, nicotine, marijuana (and other recreational drugs), and alcohol:** ✓ **“Any trouble in the past due to alcohol use?”** ○ We assume the use of alcohol in most individuals and therefore ask for an exact report as to the frequency of intake and the history of “any trouble in the past due to alcohol use”. **6.F. Participants are asked about their sleep habits:** ✓ **Question their dreaming, if they recall dreams, if there are recurring dreams, and if the dreams are mostly pleasant or unpleasant;** ○ These questions are focused on the presence of nightmares and as evidence of PTSD
**Section C. Personal History**
**7. *“Where were you born and where were you raised?”*** **This section is designed to probe long term issues: problems with mental or neurological illnesses** ○ Make inquiries into each family member’s functioning as participants often gloss over or “normalize” behaviors or proclivities of their family members, either out of ignorance or in a conscious effort to be accepted into the study ○ Major and/or frequent changes in living circumstances need to be explored (e.g., war, poverty, persecution, domestic violence, and/or potential exposure to trauma) ✓ **Note all first-degree relatives in the family of origin** ✓ **Inquire about each sibling’s health over the years, both emotionally and physically, their work, marital status, and relationship with the participant** ✓ **Briefly examine the parents’ histories and how the participant perceives them**
**8. *“Thinking about the first 12 years of your life, in a sentence or two, how was that time for you? What do you remember? What was it like?”*** **Any suggestion of difficulty is explored** **8.A. One’s school experience at elementary, middle, and high school levels is reviewed** ○ Focus on their grades, relations with teachers and peers, any special learning issues (this is where reports of heretofore undisclosed ADD and ADHD often emerge), and outside activities ○ Significant changes in school performance are explored and often reveal emotional or psychiatric concerns that were otherwise denied or overlooked ✓ **Ask specifically about their health as a child** ✓ **Were their material needs well met (food, shelter, clothes)?** ✓ **Explain any traumatic experiences in the first 12 years** ✓ **Were they ever separated from their family for any reason or period?** ✓ **What did they do after high school, either work or higher education** ***(*e.g., *“Where did you attend school, your GPA, major, length of stay, was it a positive experience, did you graduate?”*)** ✓ **Discuss the course of their work history** **8.B. Main goal: to determine ability to complete tasks and see things through, how they deal with structure and authority, and to discern how they have tended to get along interpersonally** ○ These qualities are necessary in order to fulfill their responsibilities as a study participant ○ Frequent job loss, changes, and moves are concerning and warrant further inquiry, as it suggests possible instability or unresolved personal issues ✓ **Were they able to sustain employment and if there were gaps, why?** ○ Alternatively, how do older participants/retirees manage their free time? **8.C. This section helps discern aspects of an individual’s personality—the participant’s ability to relate in a give-and-take manner, and their capacity for empathy and caring** ✓ **We need to know if there is a current significant other in the participant’s life** ○ If there is a troubled significant relationship, or if the significant other strongly objects to the participant’s participation in the study, this should be a cause for exclusion. The laboratory is not a good place for someone to be when they may be worrying or concerned about a relationship, given that communication is so curtailed (See Table 5, example 3) ✓ **How long have they known each other?** ✓ **How are they getting along?** ✓ **Are there children?** ○ If so, we need to determine if there are any disqualifying mental conditions among them
**Section D. Mental Status**
**9. Interview Behavior and Personal Appearance** **Before asking specific questions, note on a check list the behavior and personal appearance of the participant** ○ Sometimes potential participants present in offbeat, “artsy”, “goth”, or even bizarre attire; or speak unconventionally. This is not necessarily exclusionary, given that many participants come from the student population of greater Boston, and/or from diverse multicultural backgrounds. Thus, such presentations are not necessarily deemed counter-normative or problematic ✓ **How well did they relate to the clinician?** ✓ **Did they make eye contact, were they friendly and open with their responses?** ✓ **Was there any hesitation or suggestion that they were evading specific issues?** ✓ **Were they cooperative, alert, and oriented; were their thoughts well-organized?** ✓ **Were there any overt signs of anxiety, agitation, or depression?**
**10. Vegetative Functions Assessment** **Assess vegetative functions such as appetite, delving further if they reply negatively or indicate there has been notable change in their weight** ✓ **Ask for a general sense of their mood, comparing it to our own observation (note evidence of a sense of humor, the absence of which is a red flag)** ✓ **Ask about the quality of their sleep in general, how many hours do they average per night and do they feel rested and energized the following day?** ○ Extremes in the average sleep duration (less than 6 h or more than 10), absence of regularity, or excessive daytime fatigue raise concerns ✓ **Inquire about their sex drive (e.g., interest) and whether they feel it is normal**
**11. Mental Health History** ✓ **Have they ever been depressed?** ○ “Yes” is not an automatic exclusion as reactive depressions to a death or loss or life change may be acceptable so long as they were not protracted, no drug therapy or hospitalization was required, and no period ensued in which they were nonfunctional (i.e., could not get out of bed, see to their daily tasks, or maintain their personal hygiene) ✓ **Was the depressive episode self-limiting or did they seek professional help?** ○ A period of psychotherapy or counseling is not in and of itself disqualifying, so long as the other disqualifiers did not obtain ✓ **Have they ever had any suicidal thoughts?** ○ A fleeting thought with no plans, gestures, or attempts may be acceptable ✓ **Ask whether they ever had any “strange or bizarre experiences; for example, see things or hear things that weren’t really there or have any thoughts or odd feelings they could not explain?”** ○ Instances of experiences like déjà vu, for example, are not uncommon and are not of concern
**12. Outside Interests** ✓ **Ask about friendships and we ask about interests, hobbies, and ways in which they like to spend their spare time** ○ Answers may give us a clue as to: How well they will manage their time in the study where boredom can be a problem, and how they will get along with our staff with whom they will be in frequent contact

**Table 5:** Participant exclusionary examples.

Participant Exclusionary Examples	
**1. Exclusion Prior to Interview**	A 19-year-old college student had an MMPI profile with significant elevations on 4 clinical scales (i.e.,: Pd-psychopathic deviate, Pa-paranoia, Pt-psychasthenia, and Sc-schizophrenia). In a phone call after the exclusion, the profile was interpreted at the participant’s request and the accuracy of the findings was acknowledged (e.g.,: introverted, socially withdrawn, insecure, lacking self-esteem, guilt over perceived shortcomings, etc.). The participant stated a willingness to get counseling help and was responsive to an offer of a meeting with the psychologist while securing a referral.
**2. In-study Psychological Intervention**	The psychologist was called to visit with a participant after staff expressed concerns about her personal hygiene, attitudes toward some staff, and social skills. The participant revealed that she had taken offense that one of the techs had briefly looked at one of the books she was reading rather than immediately attending to her. She registered her upset by staring at a wall and being noncommunicative, which she acknowledged was a tactic she uses to register disapproval. Moreover, she admitted she is sensitive to slight and rejection and avoids direct confrontation by being passive. She responded well when told “We know these are exceptional circumstances. Not only do you have a right to express your needs at any time, we want and need your input and reactions.” Having discussed the situation, she felt freer to express her needs and sensitivities, and continued with the study until completion
**3. Dis-empaneled**	The participant was a 19-year-old female who was dis-empaneled with 11 days to go in a 32-day study. This action was precipitated by a significant drop in her mood scales, indicating she was very unhappy. After initially refusing to speak with the psychologist about the change in her mood, she did ultimately admit to missing family (who were communicating with her appropriately), but more importantly, that she had not received any return e-mail communication from her fiancé. She had a nightmare that he broke off the engagement. She knew it was only a dream but for days she did not receive an e-mail from him. Our staff contacted the participant’s sister who in turn had the fiancé write. The psychologist spoke with her after she was discharged and ascertained that she was doing well. She had never indicated at screening that she was engaged.

**Table 6:** The in-study visit process.

**To Assess How the Participant Is Faring under the Study Conditions**	
**Steps and Examples**	
**Steps**	**Examples/Explanations**
**1. Staff and Chart Review**	Before entering the participant’s room, the psychologist checks with the staff and reviews the chart to see if there are any issues of concern.
**2. Participant Interaction**	Upon entering, the psychologist will typically engage in some small talk, ask how the participant is doing, what has been the greatest challenge, have there been any surprises or unexpected aspects, did they feel well prepared?
**3. Assess Activity Level**	We want to know how they are keeping busy, and therefore try to have a discussion with them about their activities, readings, videos, etc.
**4. Assess Affects**	They are asked about their mood in general but then asked specifically about particular affects: anxiety, fear, frustration, irritation, boredom, loneliness, sadness, anger, joy, happiness, and inquire in further detail about any they endorse.
**5. Assess Sleep Quality**	We discuss their sleep quality and compare it to what they typically experience at home and inquire about dream frequency and whether their dreams are pleasant or unpleasant.
**6. Assess Appetite and Food**	We also discuss their appetite and the food. Is their appetite modified based on emotions or food quality and/or lack of choices. We ask about food because that could be a control issue and appetite changes may be associated with certain psychological issues.
**7. Assess Outside Communications**	We inquire whether they have had contacts with friends or family and how they have felt about those contacts.
**8. Assess Internal Communications**	We also discuss their relations with the nursing and technical staff, their study project leader, and the PI of the study.
**9. Assess Questions, Worries, and Concerns**	We use this visit to remind them that if anything is bothering them or causing them distress we want them to communicate with us, so we can do whatever we can within the constraints of the protocol to make them more comfortable (See Table 5, example 3). We emphasize that the worst thing in such an environment is holding back feelings or concerns.

## Appendix A

Per Partners Healthcare policies, data shared by our institution must be accompanied by an agreement outlining how the data will be used, protected, and maintained, e.g., through a formal Data Use Agreement (DUA) signed by the sharing and receiving PIs. Only deidentified data [subjective mood, responses on exit questionnaire] presented in this paper will be shared. Data sharing requests may be submitted by a nonprofit institution or entity, government agency, or other public entity. Data may be shared as part of a collaboration between the parties or for independent research by the outside party. Review and negotiation of an outgoing DUA to a nonprofit institution, government agency, or other public entity should be directed to the corresponding author, and include the following: (1) outside party entity name, Principal Investigator name and administrative contact information; (2) purpose of data exchange; (3) relevant background information; (4) information about outside party’s IRB approval for the requested data and use.
